# Use of a sample injection loop for an accurate measurement of particle number concentration by flow cytometry

**DOI:** 10.1007/s00216-024-05387-6

**Published:** 2024-06-27

**Authors:** Hye Ji Shin, Inchul Yang, Sang-Ryoul Park, Ji Youn Lee

**Affiliations:** 1 Biometrology Group, Division of Biomedical Metrology, Korea Research Institute of Standards and Science, 267 Gajeong-Ro, Yuseong-Gu, Daejeon, 34113 Republic of Korea; 2 Graduate School of Analytical Science and Technology, Chungnam National University, 99 Daehak-Ro, Yuseong-Gu, Daejeon, 34134 Republic of Korea

**Keywords:** Sample injection loop, Volumetric counting, Exhaustive counting, Number concentration, Flow cytometry, Polystyrene beads

## Abstract

Flow cytometry plays a pivotal role in biotechnology by providing quantitative measurements for a wide range of applications. Nonetheless, achieving precise particle quantification, particularly without relying on counting beads, remains a challenge. In this study, we introduce a novel *exhaustive counting* method featuring a sample loop–based injection system that delivers a defined sample volume to a detection system to enhance quantification in flow cytometry. We systematically assess the performance characteristics of this system with micron-sized polystyrene beads, addressing issues related to sample introduction, adsorption, and volume measurement. Results underscore the excellent analytical performance of the proposed method, characterized by high linearity and repeatability. We compare our approach to counting bead–based measurements, and while an approximate bias value was observed, the measured values were found to be similar between the methods, demonstrating its comparability and reliability. This method holds great promise for improving the accuracy and precision of particle quantification in flow cytometry, with implications for various fields including healthcare and environmental monitoring.

**Supplementary Information:**

The online version contains supplementary material available at 10.1007/s00216-024-05387-6.

## Introduction

Flow cytometry, a technology used to analyze the physical and chemical characteristics of individual cells or particles in a fluid sample, is commonly adopted in biology, medicine, and research to analyze and sort cells based on their properties such as size, shape, and surface markers. Recently, flow cytometry has been expanding its applications to diverse fields, such as at-line monitoring of yeast [1], detection of aerosol bacteria [2], ion concentration measurement in serum [3], and measurement of enzyme activities [4]. As it expands, the technique is often combined with other technologies, such as magnetic sensing [5] to measure a variety of analytes. One of the quantitative parameters that flow cytometry provides is particle number concentration, the precise quantification of which is essential for various industries and academic fields that utilize biological entities like cells [6–9], viruses [10, 11], bacteria [12, 13], and extracellular vesicles [14, 15] as well as non-biological entities such as microbeads and nanoparticles [16]. However, in many cases, a lack of primary measurement methods and reference materials limits the validation and evaluation of particle counting methods. To overcome these limitations, Sarkar et al. introduced an experimental design and statistical analysis that uses a dilution series of cell solutions to examine the precision and accuracy of a particular measurement procedure [17]. Although this approach can currently be employed for the validation and evaluation of particle counting methods, there is still a need for the development of primary methods for particle counting and reference materials.

The most common method for precise particle concentration measurement is to use counting beads of known concentration or count to determine the concentration of test particles [8, 18]. This method involves mixing the counting beads, which are available in either liquid or freeze-dried form, with the sample to be measured. While this method has numerous merits, it only provides a nominal value without an uncertainty estimate, and it assumes that the counting beads and test particles are evenly mixed and equally detectable in the flow cytometer, which is not always guaranteed in practical settings. Therefore, developing direct particle counting methods of high precision that do not require the use of counting beads can be useful in various applications.

To establish primary methods for particle counting, many efforts have been made by research groups in national metrology institutes. One such group has developed and improved a primary method for particle counting using a particle counter [19–21], achieving “total particle counting” based on flow cytometry. Similarly, researchers have developed a reference particle counter based on flow cytometry utilizing either impedance or optical properties of particles to accurately determine particle concentration [22, 23]. Another group has developed a primary method for particle counting using light obscuration and a dynamic imaging particle counter [24]. Furthermore, a particle counter based on flow cytometry that can directly count biological molecules such as DNA and RNA has been developed [25, 26], employing an exhaustive counting approach that analyzes all samples in the capillary with a predefined sample volume. In these works, metrological traceability was achieved by either mass or volume measurement of the injected sample.

In this work, we present a method for volumetric particle counting that utilizes a sample injection loop for “total particle counting.” To introduce a defined sample volume to the measurement unit, we employed a sample injection loop, which is commonly used in analytical instruments such as HPLC, for sample injections. While sample injection loops have been utilized in a few different flow cytometry applications, including high-throughput analysis from frequent sample injection [27] and real-time online analysis from cell cultivation reactors [28, 29], their use to achieve total particle counting remains largely unexplored, to the best of our knowledge. By employing a sample loop with appropriately assigned volumes, we can consistently load samples with reproducible injection volumes, which facilitates efficient and reliable volumetric counting. After filling with test particles, the loop is flushed with a sufficient volume of carrying buffer, and the particles in the loop are counted in the flow cell. We tested the developed method using micron-sized beads and examined its proportionality, limit of quantification, repeatability, and intermediate precision, and then compared the results with traditional counting bead–based method.

## Experimental methods

### Measurement setup

**Fig. 1:**
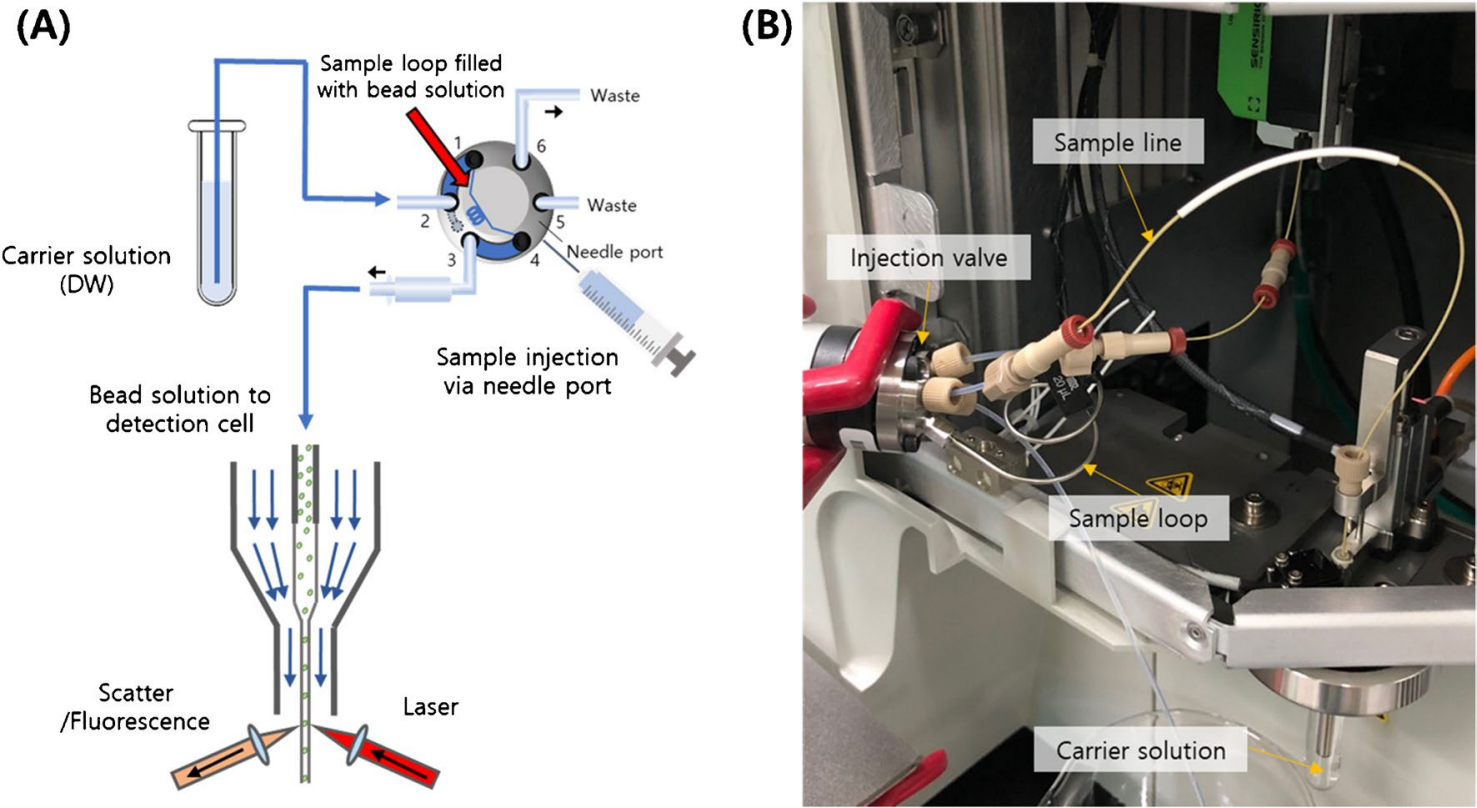
Flow cytometer configuration. **A** Schematic representation of sample loading and injection. **B** Photograph of the instrument setup showing the injection valve setting

The sample inlet configuration of FACSVerse™ (BD Biosciences) was modified with a six-port injection valve (Model 7725, Rheodyne) equipped with 20 µL sample loops (stainless steel, Rheodyne) (Fig. 1). The sample line connecting the sample port and the flow cell was cut and connected to the injection valve using appropriate fittings (Supporting information Fig. S1). Upon injection switching, a sample solution with a defined volume in the sample loop was introduced into the flow cell by a vacuum-driven carrier medium (0.2 µm − filtered deionized water in this study). For the conventional acquisition approaches, the sample loop was omitted from the sample line, and the sample solution was directly aspirated from the sample tube into the flow cell.

### Sample loop volume measurement

Prior to measurement, sample loops were dried in an oven at 50 °C overnight to remove residual moisture. To calculate the weight of water in the loop, a dried sample loop and a water-filled sample loop were weighed using an analytical balance (XS204, Mettler Toledo). The sample loop volume was then calculated by multiplying the water density by the weight of the water in the loop at room temperature. Measurements were repeated at least 5 times for each step.

### Sample flow rate measurement

For sample flow rate measurements, the flowmeter (SLI-0200HI, Sensirion) was disconnected from the flow cytometer and then connected to a computer via a USB cable. Flow rates were recorded using acquisition software (USB RS4852 Sensor View, Sensirion), and the exported data was analyzed with Excel 2019.

### Bead size measurement

The size of the polystyrene beads was measured with an inverted optical microscope (IX71, Olympus). A bead solution was loaded in a cell counting chamber, and images were collected randomly using a × 20 objective lens. A total of 142 beads from 12 images were analyzed for size measurement using ImageJ (NIH, version 1.52a).

### Bead solution preparation

#### Test bead stock solution

Polystyrene beads with a nominal size of 5.1 µm (streptavidin-coated, Spherotech) were used for the measurement. A bead stock solution was prepared at a concentration of approximately 1–2 × 10^6^ particle/mL in diluent, which consisted of deionized water (DW) containing 0.01% Tween 20. The solution was kept at 4 °C until use, and it was equilibrated to room temperature and sonicated for 5–10 min before use to prevent aggregation.

#### Dilution series test samples

For proportionality and lower quantification limit tests, dilution series were prepared using dilution fractions (DFs) of 1, 0.7, 0.5, 0.3, 0.1, 0.05, 0.03, 0.01, and 0.005, all created with a dilution buffer in triplicate. To minimize potential bias from serial dilution and to ensure the accuracy of pipetting volumes, we maintained dilution ratios below 10 (Supporting information Fig. S2). For upper quantification limit test, dilution series were prepared spanning up to 50 times the concentration of the bead stock solution, all created with a dilution buffer in triplicate. The weight of each solution was measured with an analytical balance to determine the actual dilution fraction.

#### Bead mixture with counting beads

A bead solution of DF 0.25 or 0.3 was prepared by mixing the bead stock solution with a diluent. Then, 0.5 mL of the resulting bead solution was added directly to Trucount tubes (BD Biosciences). Four tubes were combined and aliquoted for use in either sample loop or conventional measurements. The weight of the added solution was measured with an analytical balance for an accurate concentration measurement.

### Flow cytometer measurement

The bead solution was introduced into the sample loop using a syringe, ensuring that the loop was overfilled with six to eight loop volumes. Acquisition was started without previewing and carried out for 5 min. For conventional sample acquisition (without using a sample loop), the connection between the sample line and the injection valve was disconnected and reverted to its connector setting to measure the sample injected from the sample tube. The acquisition settings, including the voltage of each channel, remained consistent for all experiments except for the sample flow rate. A sample injection tube flush by applying negative pressure was performed between each acquisition. Data analysis was conducted using FlowJo (version 10.8, FlowJo LLC). For bead counting, the test bead population was gated in a scatter plot of forward scatter (FSC) versus side scatter (SSC), with debris excluded. In cases where the test beads were mixed with counting beads, we initially gated the highly fluorescent counting bead population and the non-fluorescent population in a scatter plot of the fluorescence channel (e.g., FITC or PerCP-Cy5.5) versus the SSC channel. Then the test bead population was gated, with debris excluded, in a scatter plot from the non-fluorescent population.

### Statistical analysis

All experiments were independently repeated at least in triplicate. Error bars in the graphical data represent standard deviations. A two-tailed *t*-test was used for statistical analysis in Excel 2019.

## Results and discussion

### Performance characteristics of the instrument setup

Modifying a commercial flow cytometer for the proposed sample loop − based measurement was a straightforward process (Fig. 1). The connection of the sample line tubes to the additional sample loop was made using designated parts and following the manufacturers’ instructions. Repeated disconnection and reconnection did not result in any observable variations in the experimental data (data not shown). These findings suggest that the suggested modification of the instrument setup can be adopted by commercially available flow cytometers utilizing vacuum-driven fluidics. However, it is important to verify feasibility when applying the modification to different systems.

One particular issue that arose was a reduction in the sample flow rate due to the added backpressure in the sample line caused by the insertion of an injection valve. In the given instrument, the nominal sample flow rates were 60 µL/min for medium and 120 µL/min for high flow rates. After insertion of the injection valve, however, the flow rates dropped to approximately 30 µL/min for medium flow and 61 µL/min for high flow. While this change did not affect our experiment, it may be necessary to consider increasing the vacuum strength if the reduced flow rate is not acceptable. We closely monitored whether the modified setup caused significant perturbations in the scatter histograms. While a slight shift in the forward scatter histogram was observed, overall, the histograms for both setups remained highly similar (Fig. S3A).

**Fig. 2:**
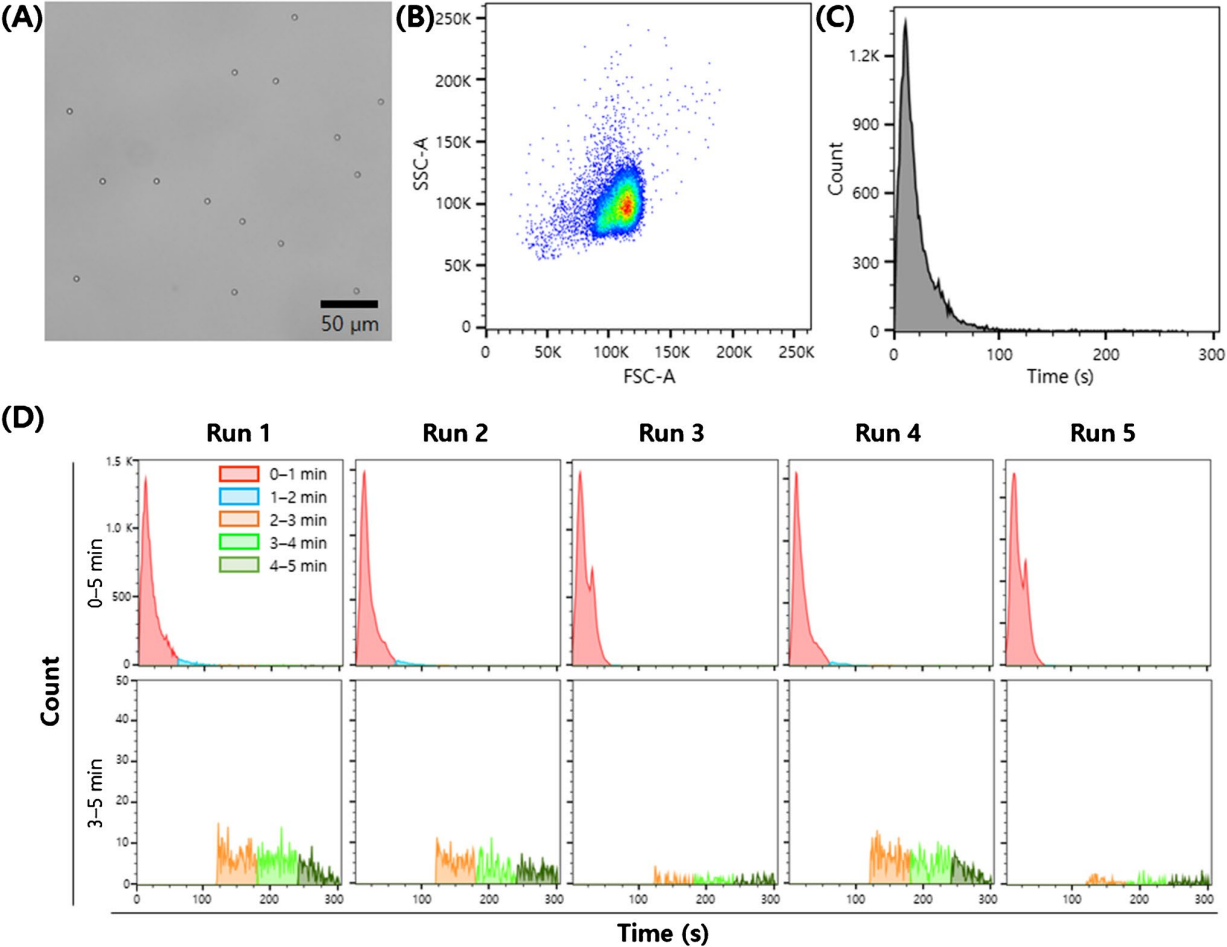
Test beads and representative measurement results. **A** Micrograph of 5 µm polystyrene beads. **B** Typical scatter plot of forward scatter (FSC) and side scatter (SSC), showing the characteristics of the test beads. **C** Typical time histogram from a 5 min acquisition of the test beads, demonstrating the distribution of events over time. **D** Time histograms of five separate runs of beads (upper panel: whole 5 min acquisition; lower panel: latter 3 min acquisition of each corresponding 5 min acquisition), showing measurement consistency

We had concerns about the thoroughness of sample introduction into the flow cytometry system to meet the principle of exhaustive counting. Following the manufacturer’s guidance, we assumed that washing the sample loop with a carrier medium at 6–10 times its volume would be sufficient to completely remove the loaded sample solution from the loop. This assumption was validated by observing a rapid rise and fall of events upon sample injection at high flow rates (Fig. 2). However, at medium flow rate, a discernable “long tail” in the time histogram indicated incomplete sample injection (Fig. S3B). Typical results demonstrated that the number of events approached the background level within 100 s, equivalent to approximately 5 times the volume of the 20 µL loop, considering the high-level carrier flow rate of 61 µL/min. Across multiple runs, it was consistently observed that the number of events reached the background level within 300 s. Therefore, a data acquisition time of 300 s at the high flow rate was chosen to ensure exhaustive counting.

**Fig. 3:**
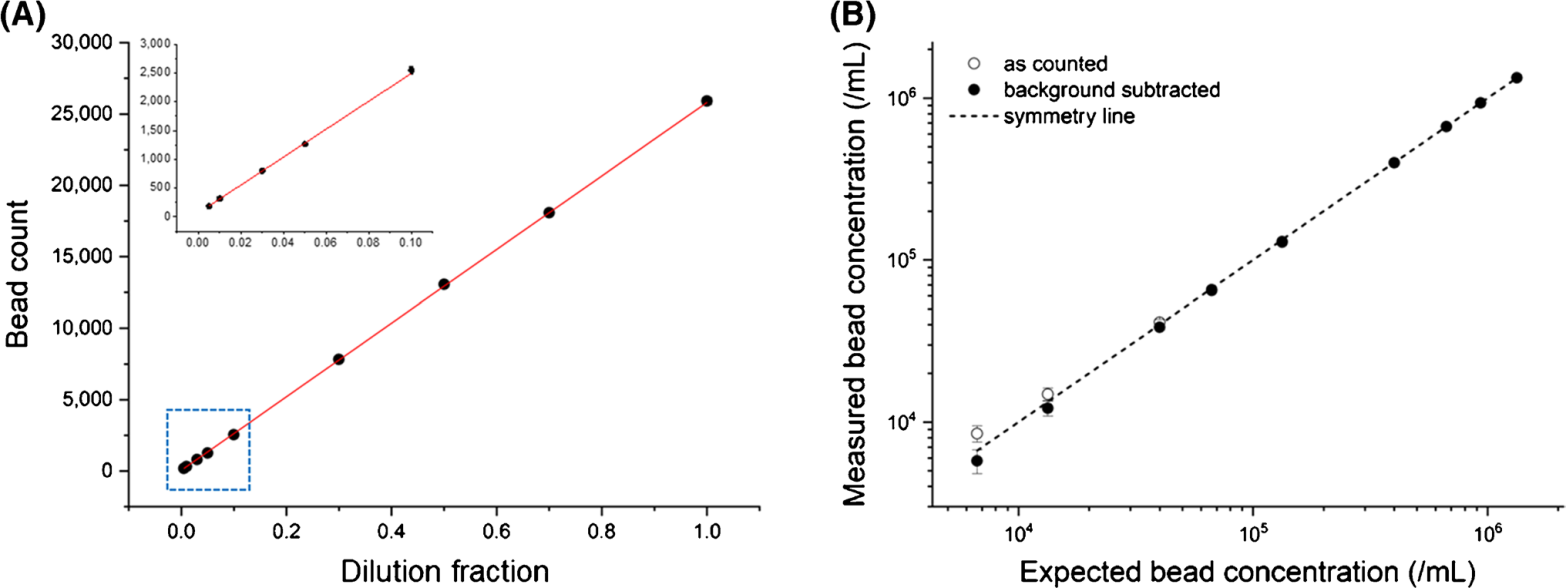
Performance of characteristics of the instrument setup. **A** Proportionality test results of dilution fractions (DFs) ranging from 0.005 to 1, with an inset highlighting the range from 0.005 to 0.1, showing a high level of linearity even with samples of low abundance. **B** Comparison of expected and measured bead concentrations both before and after background subtraction. The application of background subtraction corrects the overestimation of low-abundance samples

Another aspect of exhaustive counting that raised concern was the potential adsorption of sample material onto the inner wall of the flow channel. Fortunately, this concern was dispelled as the tested sample material did not exhibit adsorption issues. The linearity of measurements for low-abundance samples was maintained (Fig. 3), strongly suggesting that there was no sample material loss due to adsorption. Otherwise, we would have observed significant underestimations as the sample solution concentrations decreased. This conclusion aligns with the absence of significant tailing in our observations (Fig. 2 and Supporting information Table S1). However, it is important to exercise caution and consider the possibility of sample material adsorption onto the inner wall of the sample loop, depending on the properties of the sample materials.

### Sample loop volume measurement

The accuracy of the sample volume injected into the flow cytometry system plays a crucial role in determining the overall measurement precision. As we have confirmed the completeness of the injection of the loaded sample, the injected volume matches the loaded volume. As estimated via gravimetric determination, considering the density of water at a given temperature, the loaded sample volume in the 20 µL sample loop was 19.51 ± 0.01 µL. The intermediate precision of the weighing process was excellent (Supporting information Table S2), ensuring the accuracy of this measurement. As a result, we confidently adopted this value in our calculation of the measurement results.

While we verified that there was no loss of loaded sample material, a potential discrepancy remains between the actual sample volume introduced and the exact sample loop volume. This discrepancy could be attributed to the possibility of additional sample materials from components connected to the sample loop. The manufacturer guarantees no dead volume in the sample injection valve and sample loop when properly assembled. Although not substantial, there may be dead volumes in connecting regions that introduce extra sample amounts. In the current work, repeated disconnections and reconnections of the sample loop did not lead to significant changes in the measurement results, ruling out the possibility of instability in these connecting regions. Nonetheless, we are exploring alternative methods for a more precise assessment of the actually injected sample volume to validate or adjust the current sample volume.

### Analytical performance

#### Test bead solution

The test beads, 5 µm streptavidin-coated polystyrene beads, were predominantly well dispersed as single, mostly round-shaped particles, with only a few exhibiting irregular shapes (Fig. 2A and Supporting information Fig. S4A). The bead size exhibited reasonable uniformity, as evident in a typical scatter plot after a 5-min acquisition (Fig. 2B) and in the residual plot of bead diameter (Supporting information Fig. S4B). The stock solution had an approximate concentration of 1.3 × 10^6^ particles/mL and was gravimetrically diluted to prepare a series of sample solutions for testing measurement linearity. The test solution with the lowest concentration in this series (DF 0.005) contained approximately 130 beads in the 20 µL sample loop, resulting in an approximately twofold number of events compared to the background level of about 60 events. To precisely assess measurement linearity at lower concentrations and determine the reliable limit of quantification, an additional series of test solutions with low concentration levels were prepared (DFs: 0.03, 0.01, and 0.005).

#### Gating strategy

While the scatter plot of the measurements exhibited a tight clustering of events with a strong correlation between FSC and SSC channels, some scattered events were also observed (Supporting information Fig. S5). In this study, we implemented a gating strategy designed to encompass the majority of events, aligning with the principle of exhaustive counting. The validity of this strategy was assessed by comparing it to two more stringent alternative strategies using five test samples and varying the total acquisition events from 2500 to 30,000. The results demonstrated consistent levels of event inclusion regardless of the number of events. Moreover, regardless of the acquisition size, the percentage of gated events remained the same, showing that our gating strategy is acceptable for analysis.

#### Time histogram

As briefly mentioned in “Performance characteristics of the instrument setup,” time histograms exhibited sharp peaks with initial surges followed by rapid declines (Fig. 2C). Within the first minute of measurement, more than 92% of total events were acquired, and after the first 2 min, 96.5–99.5% of acquisitions occurred (Supporting information Table S1). Among the five runs conducted, three showed monotonous peaks, while the other two had minor second peaks (Fig. 2D). Interestingly, in the cases with monotonous peaks, significantly higher tails were observed compared to cases with second peaks. The elevated tail portion of the monotonous peak cases roughly corresponds to the portion of the second peak. This observation suggests that the flow path through the sample loop may vary slightly, releasing a small portion of the sample at different times and in different ways, potentially influenced by the flow channel geometry including the moving parts of the sample injection valve. However, these tails disappeared within 5 min of runtime, confirming the validity of our measurements. Across the five runs, measurement results agreed within 1.1%.

Even after 5 min, occasional events (0.7–2.3/s) were still observed, which could potentially challenge the achievement of exhaustive counting. In flow cytometry, however, background events from noise or carryover are unavoidable. Therefore, the possibility of events occurring after the initial acquisition must be compared to the background level. Under the given experimental conditions, 10 to 60 events were counted for the carrier solution (Supporting information Fig. S5A). While filtering the carrier solution with a 0.02 µm filter proved ineffective in reducing background signal, implementing particle filters in the sheath fluid supply and minimizing tubing in the sample supply line have the potential to achieve greater noise reduction. Additionally, the carryover specification for the instrument used is 0.5%, which may significantly raise the background level depending on the concentration of the previously run sample; here, the measured carryover was about 0.3% (Supporting information Fig. S6B). Subtracting typical results for a blank solution under the same conditions is necessary to prevent the overestimation of uncounted events. In practice, possibly uncounted events within a reasonable time window (e.g., 10 min) that number less than the measurement variation can be disregarded or considered as measurement uncertainty.

#### Measurement linearity and limit of quantification (LOQ)

The measurement linearity for the series of test solutions was highly satisfactory, with an R2 value of 0.999 or higher, as demonstrated in Fig. 3A. These results strongly validate the proposed measurement method. The use of a dilution series for measurements, such as with cells [17], bacteria [12], and viruses [10], has been employed to assess the quality of bioparticle counting. In particle counting, a proportionality test using a well-prepared multiple-point dilution series serves as a method to evaluate the accuracy of the measurement method [17]. The smaller the deviation in proportionality within the measurement range, the more reliable the method.

The excellent measurement linearity did show a slight upward skew for the test solutions at lower concentration levels (DFs: 0.05, 0.03, 0.01, and 0.005). However, no significant underestimation was observed at the lower concentrations, strongly indicating no significant loss of sample material along the sample line. Such losses might be caused by dead volumes or sample material adsorption on the inner wall. Instead, overestimations occurred unless background events were subtracted. While not abundant, the contribution of background events becomes notable for lower-concentration samples. For example, the number of expected events for the DF 0.005 test solution was 130, which is not significantly higher than the background events ranging from 10 to 60. Background subtraction effectively improved linearity down to the DF 0.005 test solution (Fig. 3B and Supporting information Fig. S7).

The LOQ was calculated from the standard deviation of blank samples (filtered DW), which was 14.1. This resulted in an LOQ of 141 counts for the 20 µL sample loop, corresponding to 7.2 × 10^3^/mL. This concentration is a similar concentration to the lowest sample tested, DF 0.005. However, the variability in the results for the DF 0.005 and DF 0.01 test solutions remained relatively high, with coefficients of variation (CV) exceeding 10%. In contrast, the CV was significantly reduced to below 5% for the DF 0.03 test solution. Therefore, we concluded that the DF 0.03 test solution represents the lower limit of reliable quantification. This represents approximately 800 counts for the 20 µL sample loop, corresponding to a bead concentration of 4 × 10^4^/mL. We further investigated the upper limit of the working range, observing a considerable deviation in measured bead concentration from the expected linear relationship above 2 × 10^7^/mL (Supporting information Fig. S8). This deviation is likely attributable to coincidence loss.

### Repeatability and intermediate precision

**Fig. 4:**
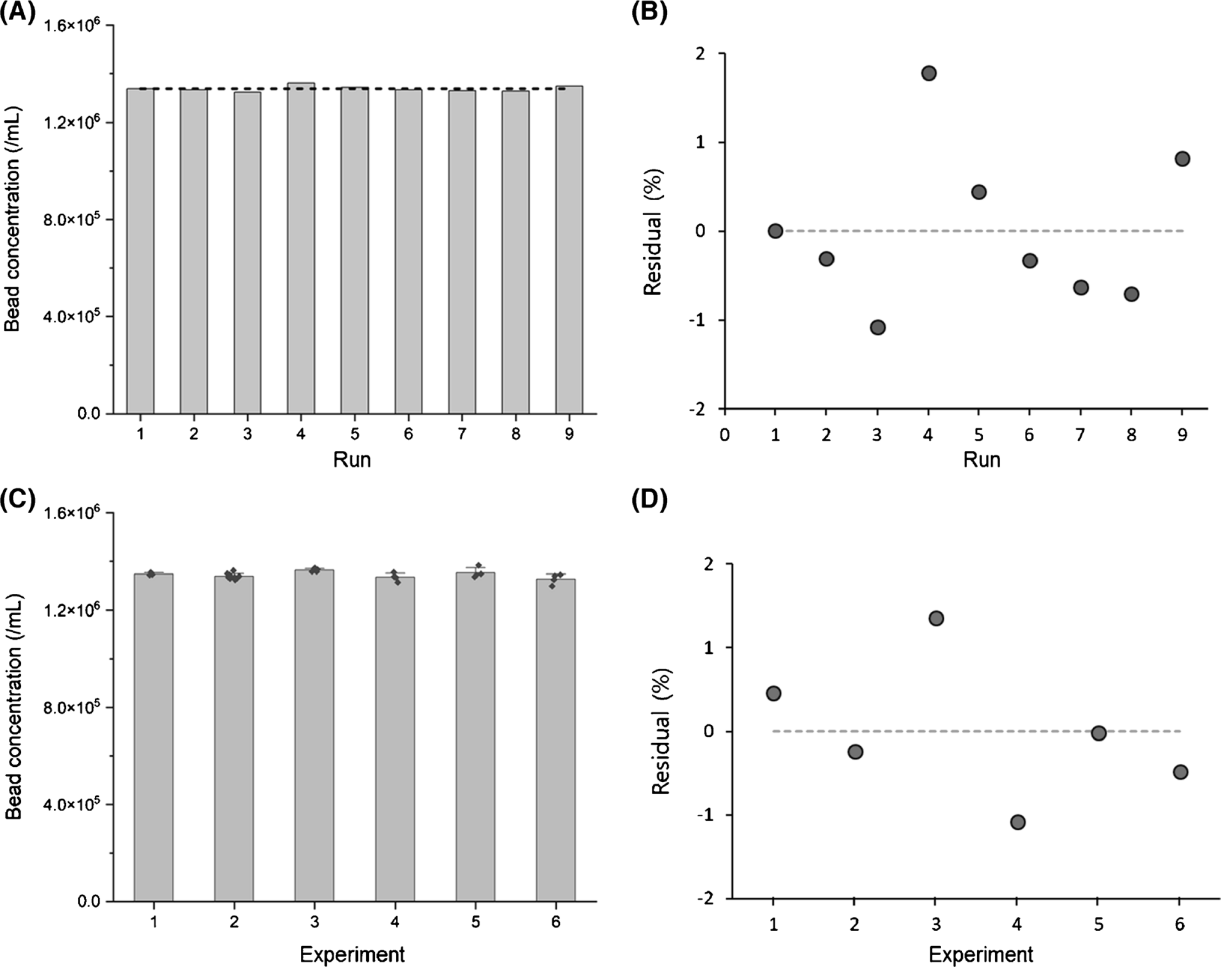
Repeatability and intermediate precision. **A** Repeatability demonstrated through nine repetitive runs of a bead stock solution. **B** Residual plot showing minor deviations among runs. **C** Intermediate precision illustrated by six repetitive experiments on different days. **D** Residual plot indicating minute deviations among experiments

Measurement repeatability and intermediate precision of the proposed method were assessed using the bead stock solution. For nine consecutive measurements, the CV was 0.89%, and the residual plot indicated deviations within 2% for all data points (Fig. 4A and B). Intermediate precision was also satisfactory, with deviations within 2% for six measurements (three replicates each) on different days (Fig. 4C and D). As mentioned above, it is common for repeatability and intermediate precision to degrade for low-level samples. However, in this study, the CVs remained below 2% for the DF 0.1 test solution, equivalent to approximately 1.3 × 10^5^/mL (2700 counts with a 20 μL sample loop).

### Method validation

To validate the developed sample loop–based particle counting method, we initially tested it with a Trucount tube, supposedly containing approximately 50,000 counting beads in freeze-dried form within a tube. We prepared a bead solution at a concentration of around 1 × 10^5^/mL and compared two sets of bead counts: the expected count calculated based on the nominal value provided by the manufacturer, and the measured count obtained using the sample loop setup. Across three independent experiments each comprising five runs, our measured value consistently fell slightly below the nominal value, ranging from 95.4 to 104.1%, with an average of 98.7% (Supporting information Fig. S9). This observation agrees with the report by Stebbings et al., which indicates that the number of beads in Trucount tubes tends to be lower than the nominal value [9].

**Fig. 5:**
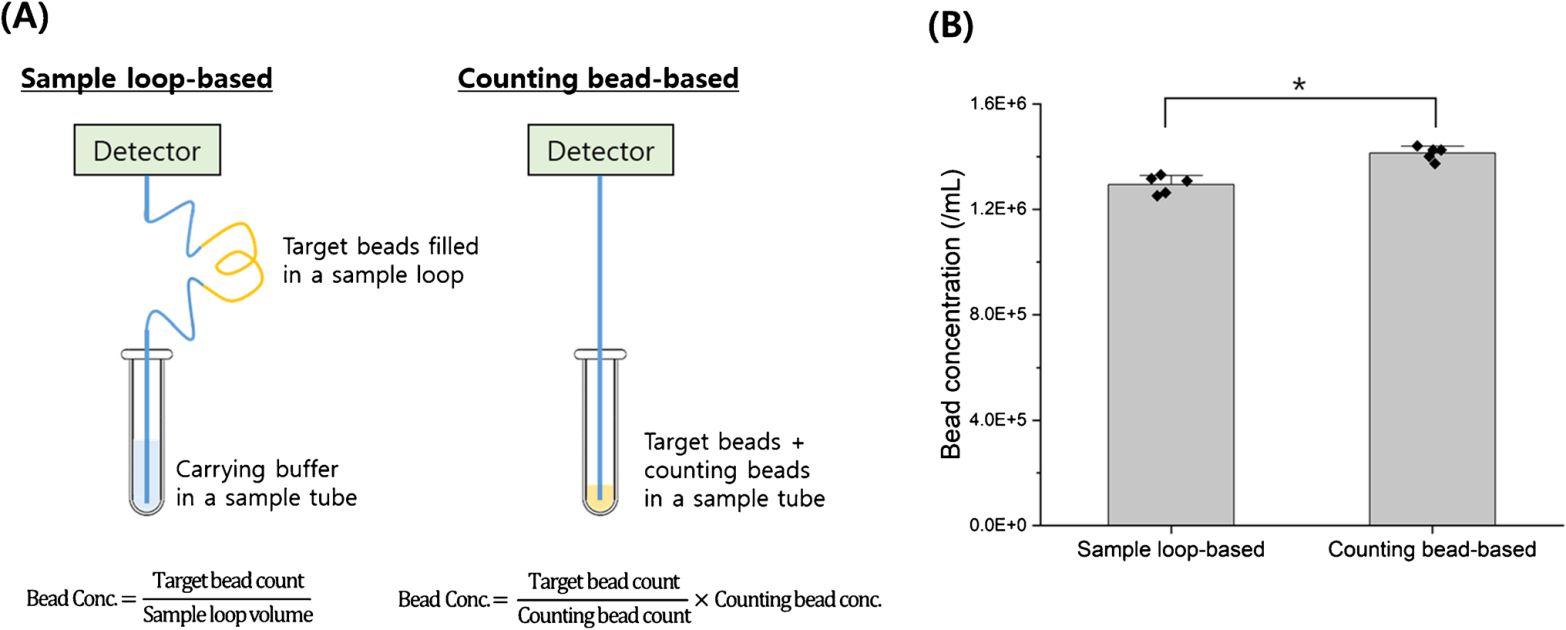
Method comparison. **A** Schematic representation of sample loop − based and conventional counting bead − based measurements, and **B** comparison results (*p > 0.05)

We then performed a comparison experiment between sample loop–based and counting bead–based measurements, as illustrated in Fig. 5A. Counting bead–based measurement has been employed for the precise quantification of blood cells [30, 31], bacteria [32], and microspheres [33]. We adjusted the reconstitution volume and DF of the test solution to ensure that no specific population dominated the acquisition, thus securing reliable measurements. We used a test bead solution of DF 0.25 or 0.3, and later applied the actual DF obtained by weight measurement to calculate the stock bead solution concentration. While the results from five independent sets of experiments were reproducible, the average value of the sample loop–based measurement was 8.4% lower than that of counting bead–based measurement, as depicted in Fig. 5B (raw data in Supporting information Table S5). This difference was larger than the simple CV of 2–2.4%. However, it would become insignificant if other sources of uncertainty, such as the variability in Trucount bead count and gating strategy, were considered. Uncertainty of the bead count in Trucount tubes is typically 2–6%, and uncertainty from variations in the stringency of the gating strategy can be up to 4%, resulting in expanded uncertainty of about 8.2–10.2%. We plan to conduct a more rigorous comparability assessment in the near future as we work to reduce those sources of uncertainty. For the time being, we assume that the proposed method is comparable to the conventional counting bead–based method within a relatively larger overall measurement uncertainty.

## Conclusions

In this study, we have presented a robust “exhaustive counting” method for absolute quantification in flow cytometry using a sample loop–based injection system. The modification of a commercial flow cytometer for this proposed method offers the potential for widespread adoption by operators. Our comprehensive analysis demonstrated the excellent performance characteristics of this setup, including a thorough sample introduction and the absence of significant adsorption issues. The analytical performance of the method was found to be excellent, with high linearity and intermediate precision. The results indicate that the proposed approach is comparable to counting bead–based measurements and offers a reliable means of quantifying particles. We aim to expand the applicability of the proposed counting approach to particles of various sizes. Unlike the counting bead − based method, which determines particle concentration relative to counting beads, our method pursues exhaustive counting and is ideally unaffected by the characteristics of the beads (e.g., size) in measuring particle number concentration. Therefore, we believe that our approach will serve as a good complementary method to other particle counting techniques. We anticipate that sample loop–based counting could be a valuable addition to the field of flow cytometry and are now working on expanding its applicability to various materials.

### Supplementary Information

Below is the link to the electronic supplementary material.

Supplementary file1 (PDF 708 KB)
