# Supplementary material for: Use of a sample injection loop for an accurate measurement of particle number concentration by flow cytometry
Source: Anal Bioanal Chem. 2024 Jun 27;416(20):4481–90. doi: 10.1007/s00216-024-05387-6 (PMC11294435; doi:10.1007/s00216-024-05387-6)
Supplement: Supplementary file 1 — Supplementary file1 (PDF 708 KB) [file 216_2024_5387_MOESM1_ESM.pdf]

Supporting Information

Use of a sample injection loop for an accurate measurement of particle number concentration by flow cytometry

Hye Ji Shin[1, 2], Inchul Yang[1], Sang-Ryoul Park[1*], and Ji Youn Lee[1, 2*]

[1]Biometrology Group, Division of Biomedical Metrology, Korea Research Institute of Standards and Science, 267 Gajeong-ro, Yuseong-gu, Daejeon 34113, Republic of Korea

[2]Graduate School of Analytical Science and Technology, Chungnam National University, 99 Daehak-ro, Yuseong-gu, Daejeon 34134, Republic of Korea

*Corresponding authors

E-mail: srpark@kriss.re.kr (SRP), jylee@kriss.re.kr (JYL)

For Peer Review

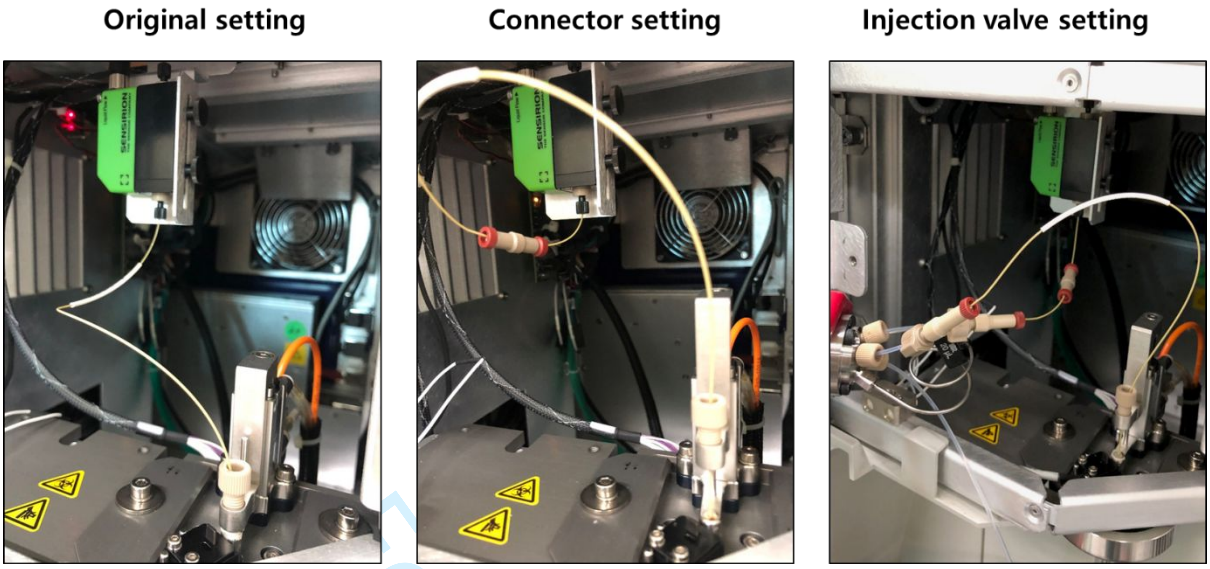

Figure S1. Modification of the flow cytometer configuration. In a conventional setting, the sample line is directly connected from the sample injection tube to a flow cell through a flowmeter. In the connector setting, the sample line is cut near the flowmeter and connected with fittings. In the injection valve setting, each end of the connection part is connected to an injection valve that is equipped with a sample loop.

For Peer Review

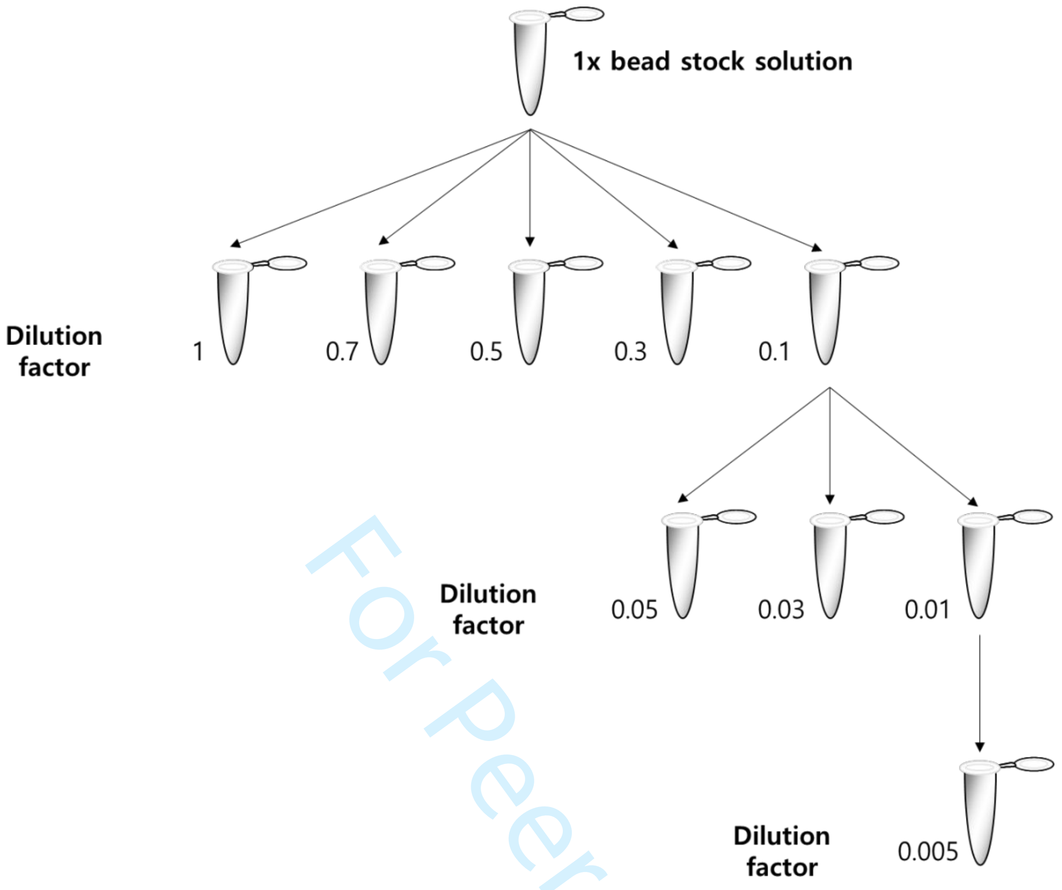

Figure S2. Dilution strategy for preparing a dilution series from 1x to 0.005x. To minimize potential bias from serial dilution and to ensure the accuracy of pipetting volumes, a three-step dilution was performed, with dilution ratios maintained below 10 for each step. Each number represents the dilution factor for the bead solutions used to measure linearity and limit of quantification.

For Peer Review

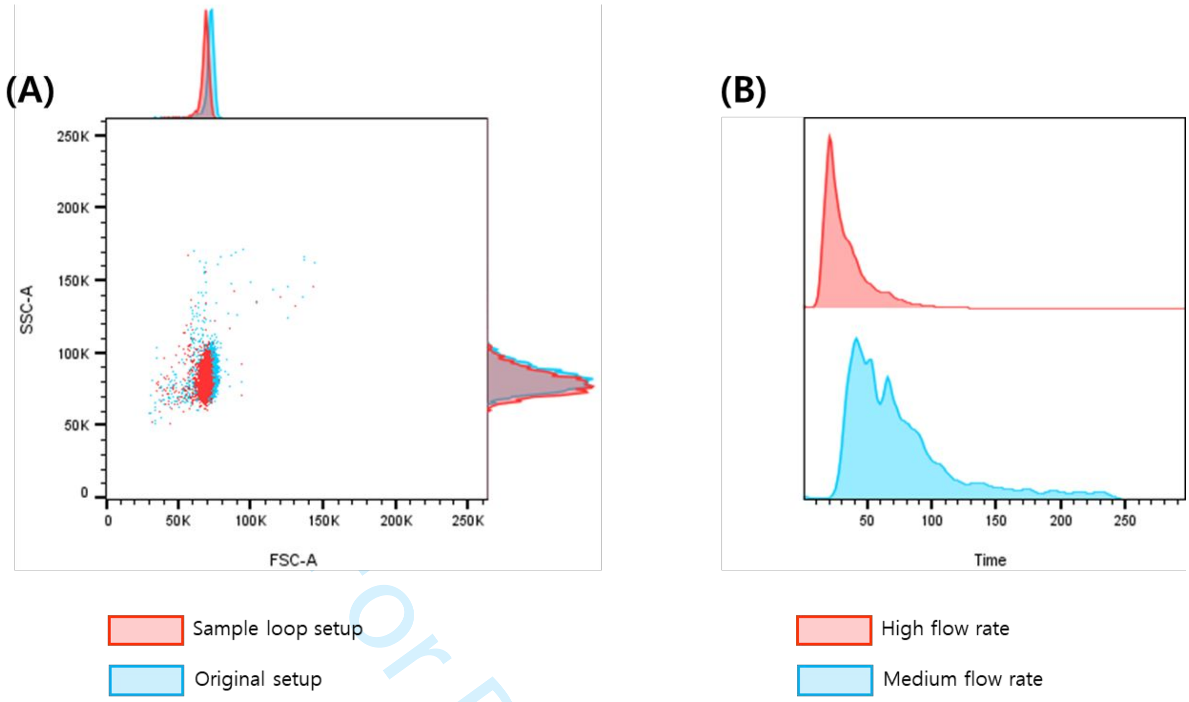

Figure S3. Performance characterization of the instrument setup with 5 µm polystyrene beads. (A) Comparison of scatter histograms obtained with different setups. (B) Comparison of time histograms obtained with different flow rates.

For Peer Review

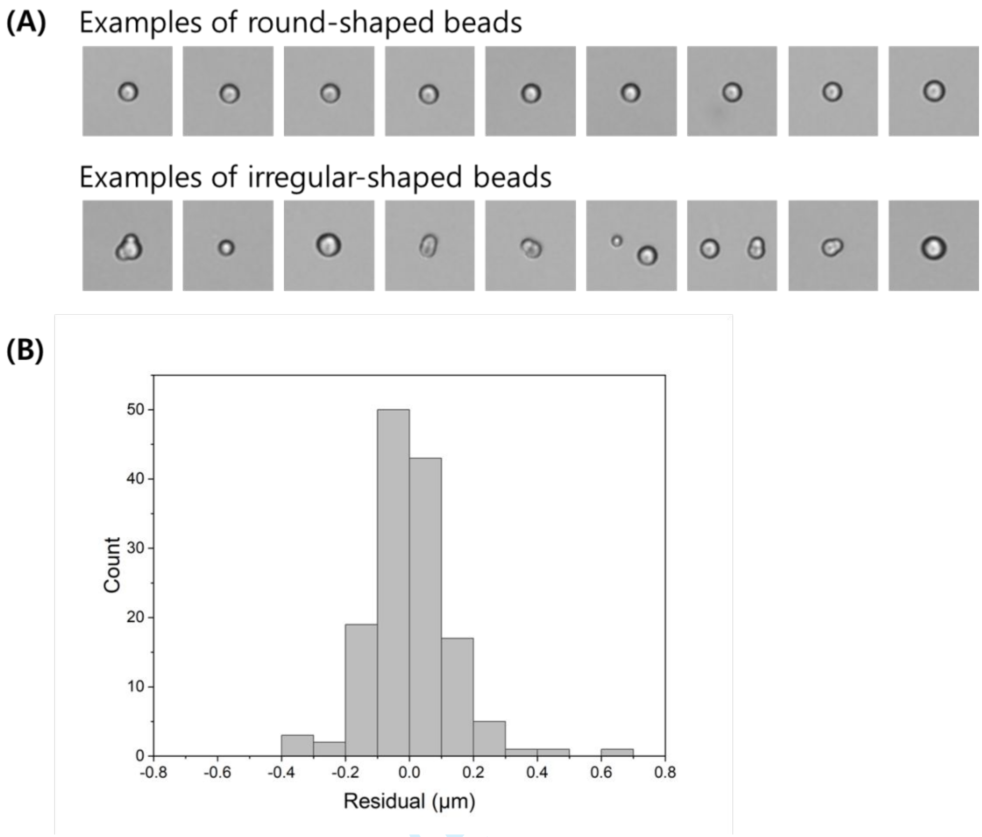

Figure S4. Shape and size uniformity of test beads. (A) Images of example test beads, showcasing both round and irregular shapes. (B) Histogram plot displaying the distribution of diameter residuals among the test beads, highlighting their size uniformity.

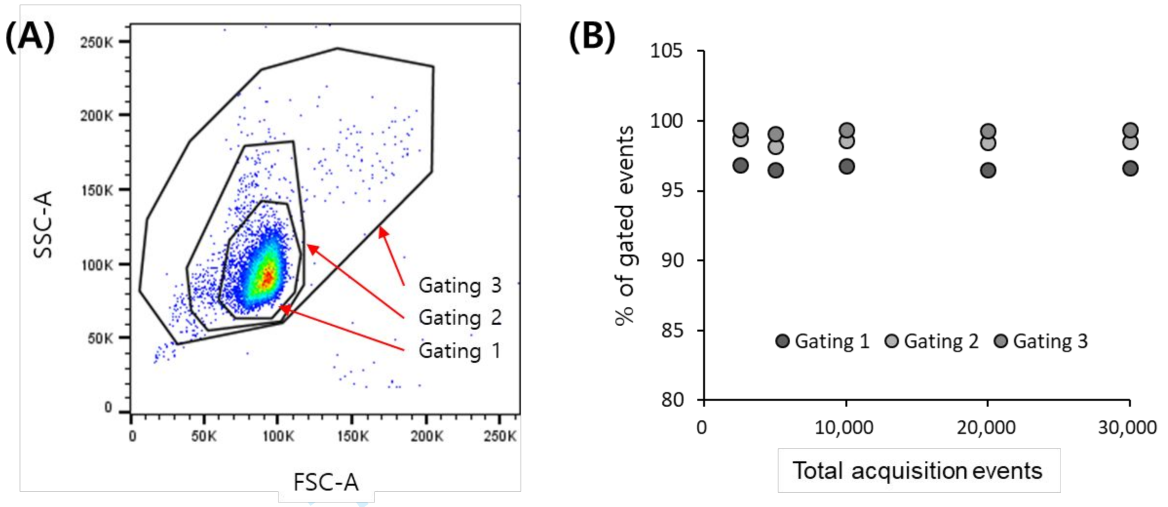

Figure S5. Gating strategy for data analysis. (A) Three different gating approaches were employed for data analysis. Gating 1 was the most stringent, aimed at including high-frequency events. Gating 2 served as an intermediate approach, capturing events near the dense population. Gating 3 was the most inclusive, encompassing the majority of events. (B) The percentage of gated events did not vary according to the number of acquisition events. The difference in the percentage of gated events among the different gating approaches varied within 4%, so we used the most inclusive gating for the analysis.

For Peer Review

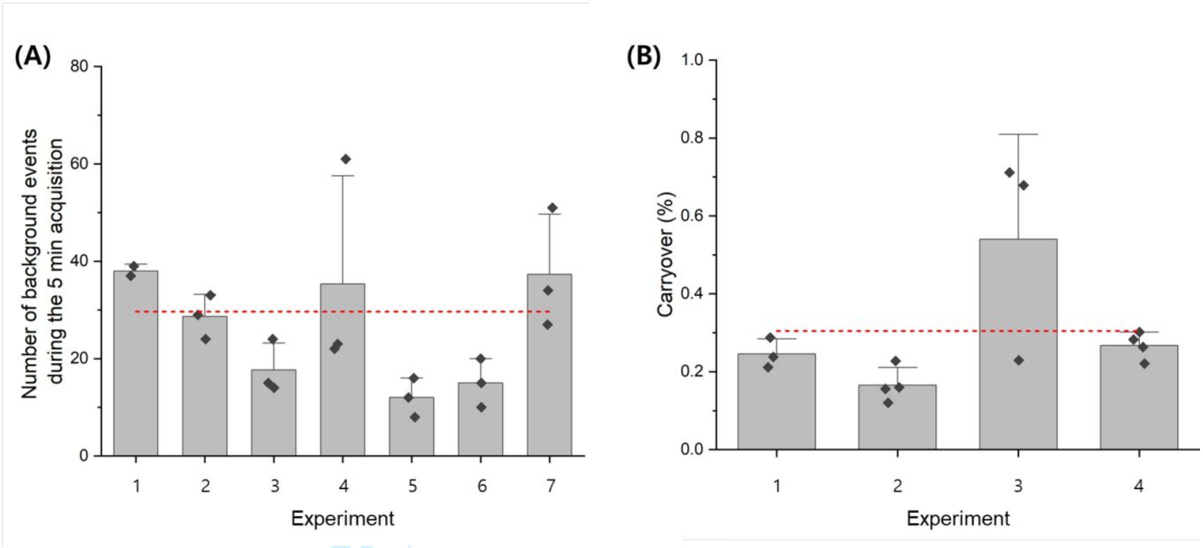

Figure S6. Characteristics of the instrument setup. (A) The background level with deionized water (DW) injection ranged from 10 to 60 events for a 5 min acquisition. (B) Carryover between samples was predominantly less than 0.5%, which complies with the technical specifications of the instrument.

For Peer Review

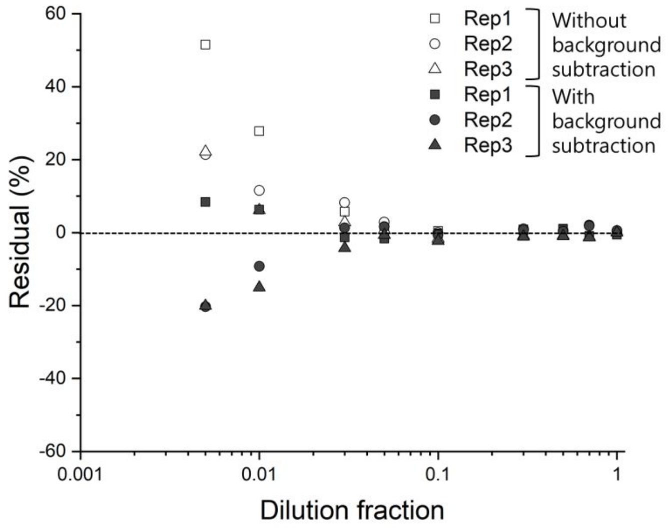

Figure S7. Measurement variability with decreasing test bead abundance. A reduced test bead abundance led to increased measurement variability. Without background subtraction, lower abundance samples were overestimated, which was corrected by background subtraction. However, significant variability remained.

For Peer Review

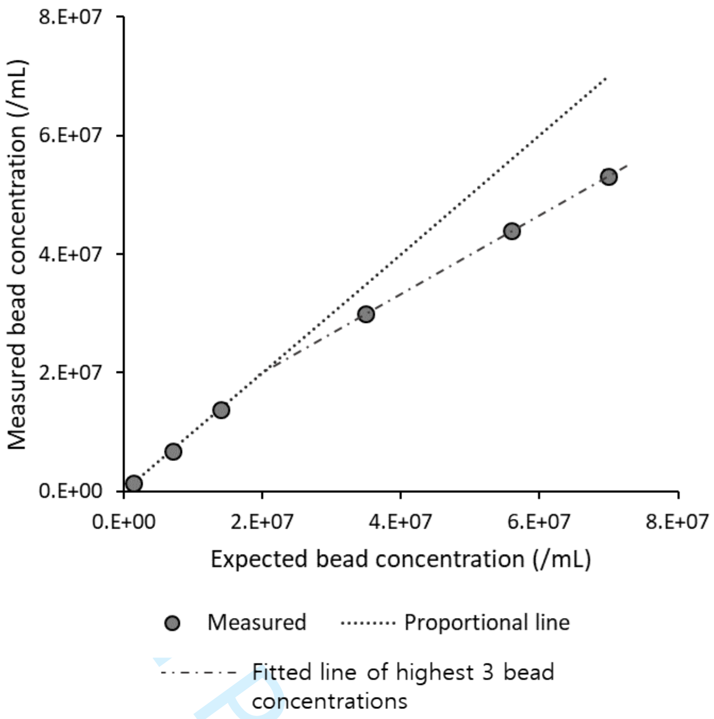

Figure S8. Upper limit of quantification. A considerable deviation in measured bead concentration from the expected linear relationship was observed at higher bead concentrations.

Peer Review

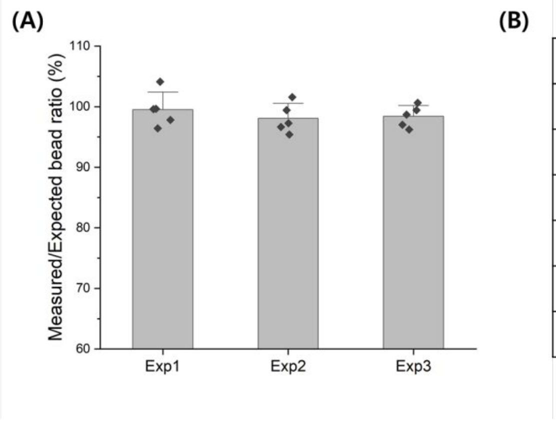

(B)

	Exp 1		Exp 2		Exp 3	
**Run**	**Expected**	**Measured**	**Expected**	**Measured**	**Expected**	**Measured**
**1**		1894		1823		1886
**2**		1979		1900		1854
**3**	1901	1833	1911	1847	1894	1900
**4**		1859		1859		1923
**5**		1893		1941		1839

Figure S9. Validation of sample loop–based particle counting with Trucount^{TM} beads. (A) A comparison of the expected to measured bead count demonstrated a high correlation between the two and (B) corresponding raw data.

For Peer Review

Table S1. Raw data for Fig. 2D

	Time (min)	Run 1	Run 2	Run 3	Run 4	Run 5	Avg.	Std. dev.	Composition (%)
	**Total**	26079	25875	26625	26268	26373	26244	285	100.00
Bead count	**0–1**	24030	24035	26270	24500	26012	24969	1090	95.14
	**1–2**	1166	1145	198	962	224	739	489	2.82
	**2–3**	372	310	47	359	52	228	165	0.87
	**3–4**	338	200	54	289	35	183	136	0.70
	**4–5**	170	172	52	155	42	118	65	0.45

For Peer Review

Table S2. Raw data for the weight measurement for the sample loop volume estimation (unit: g)

W_{loop}			$W_{loop+DW}$		$W_{DW} = W_{loop+DW} - W_{loop}$	
**Exp1**	**Exp2**	**Run**	**Exp1**	**Exp2**	**Exp1**	**Exp2**
9.09932	9.09869	**1**	9.11888	9.11831	0.01956	0.01962
		**2**	9.11894	9.11808	0.01962	0.01939
		**3**	9.11886	9.11807	0.01954	0.01938
		**4**	9.11869	9.1183	0.01937	0.01961
		**5**	9.11860	9.11808	0.01928	0.01939
		**6**	9.11865	—	0.01933	—
		**Average**	9.11877	9.11877	0.01945	0.01947
		**Std. dev.**	0.00014	0.00014	0.00014	0.00014
		**RSD (%)**	0.0015	0.0015	0.72	0.72
		**Average**			**0.01946**	
		**Std. dev.**			0.00002	
		**RSD (%)**			0.09%	

W_{loop} : weight of dried sample loop

$W_{loop+DW}$: weight of water-filled sample loop

W_{DW}: $W_{loop+DW} - W_{loop}$: weight of filled water

For Peer Review

Table S3. Raw data for the proportionality test and limit of quantification in Fig. 3. Measured bead counts are averaged from three replicates.

Dilution fraction (DF)	Anticipated bead count	Measured bead count		
		Average	Standard deviation	Relative standard deviation
**1**	26150	26054	141	0.54%
**0.7**	18305	18234	345	1.89%
**0.5**	13075	13005	84	0.65%
**0.3**	7845	7767	69	0.89%
**0.1**	2615	2526	38	1.52%
**0.05**	1308	1279	23	1.81%
**0.03**	785	806	31	3.89%
**0.01**	262	291	25	8.63%
**0.005**	131	166	19	11.33%

For Peer Review

Table S4. Raw data for the intermediate precision measurement in Fig. 4

Experiment		1	2	3	4	5	6
Run	**1**	1343209	1338630	1357663	1333931	1342799	1298052
	**2**	1345669	1334376	1366325	1313326	1383496	1339467
	**3**	1354741	1324022	1357868	1354895	1348078	1324141
	**4**		1362310	1372578	1336443	1336084	1344336
	**5**		1344422	1363608			
	**6**		1334068				
	**7**		1330121				
	**8**		1329045				
	**9**		1349496				
	**Average**	1347873	1340752	1363608	1334649	1352614	1326499
	**Std. dev.**	6074	14171	7214	17013	21165	20826
	**RSD (%)**	0.45%	1.06%	0.53%	1.27%	1.56%	1.57%

For Peer Review

Table S5. Raw data for a method comparison experiment (for Figure 5)

		Sample loop-based	Counting bead-based
**Average**	Exp. 1	1251907	1373434
	Exp. 2	1263788	1400938
	Exp. 3	1331589	1441312
	Exp. 4	1307475	1425340
	Exp. 5	1316432	1425419
**Std. Dev.**	Exp. 1	43492	6342
	Exp. 2	116503	38731
	Exp. 3	11668	57325
	Exp. 4	21694	43728
	Exp. 5	40550	45349

For Peer Review
